# Advances in Radiotherapy for Prostate Cancer Treatment

**DOI:** 10.1155/2016/3079684

**Published:** 2016-07-25

**Authors:** Tarun Podder, Daniel Song, Timothy Showalter, Luc Beaulieu

**Affiliations:** ^1^Case Western Reserve University, Cleveland, OH 44106, USA; ^2^Johns Hopkins University, Baltimore, MD 21231, USA; ^3^University of Virginia Medical Center, Charlottesville, VA 22903, USA; ^4^Laval University, Quebec, QC, Canada G1R 2JC

Prostate cancer is one of the most common cancers in men, and there are a number of management options for localized disease, such as active surveillance, radical prostatectomy, chemotherapy, intensity-modulated radiotherapy, radiofrequency ablation, cryotherapy, stereotactic ablative radiotherapy, particle therapy, brachytherapy, and an emerging range of focal therapies. Among these options, multiple competing treatment modalities can be equally effective for any particular patient, with desirable clinical outcome. However, over the years, remarkable developments and improvements have been observed in regard to clinical practice. These changes are compounding effects originating from scientific findings, technological advancements, socioeconomic demands, patient's expectations, and improvements in clinical understanding. In this special issue, we have articles addressing recent advances in treatment, clinical outcome, and scientific findings related to radiation therapy (RT) for prostate cancer.

Depending on the stage of cancer, there can be single-modality or multimodality RT options. Multimodal image-guidance is very important for the latest prostate radiation therapy options in the era of precision medicine. For high-risk prostate cancer, long-term androgen deprivation therapy and dose escalated RT together comprise a standard of care combination approach. Evaluation of the latest evidence for management of high-risk prostate cancer, including consideration of emerging RT modalities such as moderately hypofractionated RT or stereotactic body radiation therapy (SBRT), is very useful. Along with the improvement of treatment techniques, this special issue includes emphasis on how improvement in imaging, especially multimodal MRI, plays an important role in prostate cancer diagnosis, staging and precise delineation of target volume, and treatment delivery. Precise target delineation is very critical for partial prostate treatment, which is also gaining popularity.

For early stage prostate cancer, low-dose-rate (LDR) brachytherapy is considered an effective treatment option. However, delivering LDR seeds precisely and retaining seeds in their planned locations can be challenging. For patients with biochemical recurrence due to underseeded areas after primary brachytherapy, salvage brachytherapy can be a successful procedure, with some increased side effects.

Finally, this issue includes emphasis on minimizing the side effects of prostate RT. Researchers have described the metabolic mechanisms by which increased activity levels and exercise can help to improve both outcomes for men treated for prostate cancer while lowering the side effects of treatment. It is important for clinicians to encourage and give patients support for physical activity during and after treatment for prostate cancer. A systematic review of late gastrointestinal toxicity has indicated that acute toxicity is significantly associated with late toxicity. It has been suggested that acute gastrointestinal toxicity could be considered as a predictive marker for increased risk of moderate to severe proctitis and to identify patients who may benefit from additional medical interventions.

Overall, the articles in this issue provide an overview of the tremendous recent improvements in technology and clinical understanding of prostate cancer and suggest future directions for improving patient outcomes after prostate cancer treatment.

*Tarun Podder* *Tarun Podder* *Daniel Song* *Daniel Song* *Timothy Showalter* *Timothy Showalter* *Luc Beaulieu* *Luc Beaulieu*

